# Assessing the Usability of the Automated Self-Administered Dietary Assessment Tool (ASA24) among Low-Income Adults

**DOI:** 10.3390/nu11010132

**Published:** 2019-01-10

**Authors:** Julia Kupis, Sydney Johnson, Gregory Hallihan, Dana Lee Olstad

**Affiliations:** 1 W21C Research and Innovation Centre, Cumming School of Medicine, University of Calgary, Calgary, AB T2N 4Z6, Canada; jnkupis@ucalgary.ca (J.K.); sydney.johnson@ucalgary.ca (S.J.); gmhallih@ucalgary.ca (G.H.); 2 Department of Community Health Sciences, Cumming School of Medicine, University of Calgary, Calgary, AB T2N 4Z6, Canada

**Keywords:** usability, human factors, dietary assessment, Automated Self-Administered Dietary Assessment Tool (ASA24), 24-h dietary recall, low socioeconomic status

## Abstract

The Automated Self-Administered Dietary Assessment Tool (ASA24) is a web-based tool that guides participants through completion of a 24-h dietary recall and automatically codes the data. Despite the advantages of automation, eliminating interviewer contact may diminish data quality. Usability testing can assess the extent to which individuals can use the ASA24 to report dietary intake with efficiency, effectiveness, and satisfaction. This mixed-methods study evaluated the usability of the ASA24 to quantify user performance and to examine qualitatively usability issues in a sample of low-income adults (85% female, 48.2 years on average) participating in a nutrition coupon program. Thirty-nine participants completed a 24-h dietary recall using the ASA24. Audio and screen recordings, and survey responses were analyzed to calculate task times, success rates, and usability issue frequency. Qualitative data were analyzed thematically to characterize usability issues. Only one participant was able to complete a dietary recall unassisted. We identified 286 usability issues within 22 general usability categories, including difficulties using the search function, misunderstanding questions, and uncertainty regarding how to proceed to the next step; 71.4% of participants knowingly misentered dietary information at least once. Usability issues may diminish participation rates and compromise the quality of ASA24 dietary intake data. Researchers should provide on-demand technical support and designers should improve the intelligence and flexibility of the ASA24’s search functionality.

## 1. Introduction

Accurate and detailed characterization of dietary intake is essential in nutrition research. However, assessment of intake is challenging because researchers must typically rely on self-reported methods, including 24-h dietary recalls, food-frequency questionnaires, and food records [1]. Dietary intake data collected in this way are subject to bias stemming from systematic measurement error, which may result in inaccurate and imprecise estimates of dietary intake [2,3,4]. Compared to other self-reporting instruments, 24-h dietary recalls capture intake with less bias, and have, therefore, emerged as a preferred means of dietary assessment [3,4]. Until recently, dietary recalls were typically conducted in person or via telephone using the computer-assisted Automated Multiple Pass Method. In this method, trained interviewers use a five-step multiple-pass approach to obtain details of all foods consumed from midnight to midnight the previous day, with manual data entry and auto-coding for most foods [1]. The time and expense of collecting and analyzing data gathered in this way made 24-h recalls impractical for use in most large, community-based studies. 

In 2009, the National Cancer Institute released the Automated Self-Administered 24-h Dietary Assessment Tool (ASA24): an automated, self-administered web-based tool that guides participants through completion of a 24-h dietary recall and automatically codes the data [5]. The ASA24-Canada was released in 2014 and updated in 2016 [6]. The self-administered and automated nature of the ASA24 has made collection of 24-h dietary recall data feasible in large studies. Nevertheless, despite its apparent advantages, eliminating contact with an interviewer may introduce additional challenges and different sources of error, with potential implications for the quantity and quality of the data that are collected.

A limited number of studies have discussed the usability of the ASA24 in relation to the quality of dietary intake data collected [2,7]. Although intakes were on average underreported on the ASA24 compared to more objective measures [2,7], the ASA24 nevertheless appeared to provide reasonable estimates of dietary intake comparable to, or better than, those derived from other self-report methods [2,7,8,9]. Usability related to the acceptability and feasibility of use of the ASA24 has been examined through retrospective questionnaires and by examining completion rates and intake data. Findings suggest that lack of internet access and/or lower levels of computer literacy may limit participation by some populations such as older adults, racial minorities, and those with lower levels of education [8]. Participant workload may also pose a barrier with some participants finding it simpler and faster to interact with an interviewer rather than to search for and select foods themselves [8]. Other limitations include difficulties finding exact matches for foods entered in search bars and a resulting tendency to select items that appear near the top of the list [7,10]. Overall, varying levels of receptivity to using the tool have been reported [8,9,10]. 

Comparing intake data generated by the ASA24 to data generated by other measures can allow quantification of measurement error. However, such assessments cannot identify critical points in the reporting process where errors often originate. Similarly, although retrospective reports can identify socio-demographic characteristics of participants who find the ASA24 challenging to complete, the comprehensiveness, accuracy, and ultimate usefulness of these data is diminished by a reliance on participants’ ability to recall specific details of difficulties they encountered during a lengthy reporting process (~40 min [9,10]) and by the questionable assumption that participants can accurately pinpoint the errors they made as well as their cause. Moreover, the common use of closed-ended questions to query participants limits the ability to describe specific qualitative aspects of usability in detail. 

In this respect, the science of Human Factors may offer new avenues for understanding how human–system interactions contribute to dietary measurement error, particularly those associated with novel technology-enabled assessments such as the ASA24. Human Factors as a science is concerned with the interaction between people and designed systems, and how human limitations and capabilities (e.g., limitations in human working memory), and the design of those systems (e.g., the number of digits an individual has to keep in working memory to complete a task), interact. The study of usability is a sub-discipline within the Human Factors field that seeks to understand the extent to which individuals can interact with a system to achieve a desired outcome with effectiveness (i.e., the accuracy and completeness with which specified users can achieve specified goals in particular environments), efficiency (i.e., the resources expended in relation to the accuracy and completeness of goals achieved), and satisfaction (i.e., the comfort and acceptability of a system to its users and other people affected by its use) [11]. The construct of usability can also be extended to include concepts such as learnability, legibility, readability, and comprehension. A simpler way to frame the usability paradigm is to examine the relationship between usability, utility, and usefulness: a system may be technically able to deliver utility (i.e., it is technically functional) when used under perfect circumstances, but usability defines the required effort, and experience of, an individual to access that utility and ultimately interact with the system to produce a useful outcome [12].

There are a variety of methods available to evaluate software usability. The present study follows the guidelines established by the International Organization for Standardization (ISO), where usability testing of a system engages representative users to complete representative tasks within the system in order to calculate measures of efficiency, effectiveness, and satisfaction [13]. Usability testing also includes a methodology to identify usability issues and inform system design through the collection and analysis of qualitative data regarding users’ perspectives [14]. Techniques such as the “think-aloud” method encourage users to verbalize cognitive processes while interacting with the system of interest [15]. 

To our knowledge, no previous studies have involved structured usability testing of the ASA24 to obtain quantitative measures of efficiency and effectiveness, or to describe qualitative aspects of usability in detail. Therefore, the purpose of this study was to conduct a structured usability test of the ASA24 to generate quantitative measures of user performance (i.e., task success, task time, food item count, and usability issue frequency) and to examine qualitative aspects of usability (i.e., describe usability issues and user preferences) within a specific user population. The results of the usability test can provide insights that can be applied to configure and administer the ASA24 in a manner that makes the tool more usable for individuals completing a dietary recall, thereby increasing the quantity and quality of the dietary intake data that are collected.

## 2. Materials and Methods 

### 2.1. Study Design

This was a cross-sectional, mixed-methods study in which qualitative and quantitative data were collected concurrently and integrated during analysis. Mixed methods were used for purposes of complementarity to provide a comprehensive and rich understanding of usability issues [16]. This study was conducted as a pre-cursor to a larger funded study designed to investigate the impact of the British Columbia Farmers’ Market Nutrition Coupon Program (FMNCP) on the dietary intake and mental and social well-being of program participants. The FMNCP provides low-income households with 16 weeks’ worth of vouchers that can be used to purchase selected healthy foods (e.g., fruits, vegetables, nuts, seeds, legumes, meats) at participating farmers’ markets in the province of British Columbia, Canada [17]. The study was conducted in accordance with the Declaration of Helsinki and received ethical approval from the Conjoint Health Research Ethics Board at the University of Calgary (REB17-1076).

### 2.2. Screening Criteria and Recruitment

Study participants were recruited from households participating in the British Columbia FMNCP. Coupons are normally distributed via local community partner organizations in each community. Given their existing relationships with FMNCP participants, community partner organizations were asked to facilitate participant recruitment for the current study. The FMNCP manager invited approximately 100 community partner organizations to recruit participants through individual conversations as well as two broadcast emails, of which 13 agreed, with 6 ultimately enrolling participants spanning 6 different communities. Attempts were made to specifically recruit organizations working with older adults (≥60 years) as well as recent immigrants to ensure the study population was reflective of FMNCP participants who would participate in the subsequent larger study. All participating community organizations were asked to recruit study participants via posters posted on-site, broadcast or direct emails, announcements during programming, and/or in-person requests. Interested individuals were asked to sign a preliminary consent form granting the research team permission to contact them directly and conduct eligibility screening. 

Participants were deemed eligible to participate if they met the following inclusion criteria: adults (≥18 years of age), not pregnant or breastfeeding, not reporting having a cognitive disability, and able to speak, read, and write in English. Individuals without home internet/computer access were offered access to both at community partner organization sites. A total of 80 participants were screened for eligibility across the 6 participating community partner sites. Of these, 67 individuals were eligible and were invited to participate in the study, of which 51 agreed. Of those 51 individuals, 11 either cancelled or did not show up to their session and were not able to be rescheduled. One individual attempted to participate but was excluded due to technical difficulties. In total, 39 individuals participated in the study. Usability testing typically gathers in-depth data from a small number of participants (i.e., *n* = 6–8) and, therefore, our sample size of 39 divided across three groups was deemed sufficient [18].

### 2.3. Participant Consent and Compensation

Individuals who agreed to participate in the study were sent an e-mail from the research team containing a link to a web-based data collection platform and a username/login and password for the ASA24. Participants read through an online consent form embedded within the web-based data collection platform, and indicated their agreement to participate. Participants were offered $20 worth of FMNCP coupons in appreciation of their time. 

### 2.4. Online Survey Instruments

As the ASA24 is typically administered along with other multi-component questionnaires, participants were asked to complete a socio-demographic and health-related survey prior to using the ASA24 in order to approximate real-world conditions. The web-based data collection platform (SurveyMonkey 2017; San Mateo, CA, USA) guided participants through completion of a socio-demographic questionnaire and multi-item scales to assess social connectedness, perceived stress, and mental well-being, followed by the ASA24 for Canada, 2016 (National Cancer Institute 2016, Rockville, MD). Briefly, the data collection platform consisted of the following:

- *Socio-demographics:* questions to assess date of birth, gender, race/ethnicity, citizenship status, country of birth, marital status, pregnancy, perceived health status, household composition, employment status, education level, household income, receipt of social assistance, food insecurity status, language ability, and usual fruit and vegetable intake. Items were adapted from existing surveys [19,20].
- *Social connectedness:* the 20-item Social Connectedness Scale-Revised, a valid and reliable scale that reflects individuals’ perception of their closeness with others [21].
- *Perceived stress:* the 10-item version of the Perceived Stress Scale, a valid and reliable measure of the degree to which respondents perceive their lives to be unpredictable, uncontrollable, or overwhelming [22].
- *Mental well-being:* the 14-item Warwick-Edinburgh Mental Well-being Scale, a valid and reliable measure of both feeling and functioning aspects of mental well-being [23].
- *ASA24*: the ASA24 guides participants through the process of reporting dietary intake for the previous day [6]. The version used by the participants asked for a single “midnight to midnight” recall and included the “location” and “source” modules. In an initial quick list, participants select an eating occasion, indicate time of consumption, and report all foods and beverages consumed at that time. Foods and beverages can be entered by typing in specific search terms and selecting items from a returned list. Details of food types, preparation methods, portion sizes, and additions are subsequently queried during a detailed pass. The system later prompts users to recall frequently omitted/forgotten foods and to complete a final review of all items consumed.

### 2.5. Test Moderation and Procedure

A usability test moderator is a trained researcher who observes and directly interacts with participants during a usability test. Like all other researcher/participant interactions, moderators attempt to remain unbiased and neutral in their interactions. However, they also seek to elicit information from participants regarding their experiences and may provide participants with assistance to complete tasks [24]. This interaction generates valuable qualitative data, but creates a scenario that is less representative of how individuals typically interact with software under real-use conditions. In the present study, participants were sequentially assigned to complete the ASA24 in one of three “moderation groups” with varying levels of moderator involvement to account for the strengths and limitations of moderator interaction. Sequential assignment was chosen to avoid creating groups of unequal size. Table 1 provides a summary of the differences between the moderation groups that participants were assigned to. 

#### 2.5.1. Moderated and Semi-Moderated Procedure

Participants assigned to the moderated (*n* = 10) and semi-moderated (*n* = 12) groups scheduled a session to join an online meeting with one of two trained moderators who adhered to the same protocol. Participants were encouraged to participate from their computing environment of choice. During the session, participants completed the previously described survey instruments while moderators used Adobe Connect Meeting software (Adobe Systems Incorporated 2017; San Jose, CA, USA) to capture audio and screen recordings. For participants who could not provide digital audio, a telephone recording was used to capture audio data. Moderators also maintained detailed written notes of all sessions. 

The process of providing assistance to participants was formalized a priori to ensure consistent moderator–participant interaction. Participants in the moderated and semi-moderated groups were informed that they should use the ASA24 as they normally would, and that moderators could answer questions that they had while trying to complete each task. Moderators encouraged participants to resolve their own problems independently before providing assistance. (For example, if a participant stated “I do not know what to do now,” the moderator responded “Where do you think you would click on the page to proceed?”). The moderator allowed participants to experience and express difficulty and frustration until it was determined that the participant was likely to fail the task and/or withdraw from the study, at which point the moderator offered to assist the participant to continue using the platform. 

#### 2.5.2. Unmoderated Procedure

Participants assigned to the unmoderated group were split into two sub-groups. The first group of participants (*n* = 5) scheduled a time, at their convenience, to participate in the study and complete the surveys. The second group of participants (*n* = 12) received an unannounced email inviting them to complete the surveys and were given a 36-h time frame in which to do so. Limiting the time frame for survey completion was intended to minimize reactivity, where participants change their dietary intake in anticipation of having to report it [25,26]. Participants in the unmoderated group (*n* = 17) completed data collection entirely independently and had no contact with the study team. Audio and screen recordings were not collected for participants in the unmoderated group and only their survey responses were available for analysis. 

#### 2.5.3. Data Collection

The data collection procedures for each of the three moderation groups (as presented in Table 1) are described in detail below:

- Participant think-aloud: the think-aloud procedure involves asking participants to verbalize their thoughts and feelings as they use a system [15]. Participants were asked to “think aloud” as they completed the surveys, were encouraged to elaborate on difficulties encountered (both conceptual and technical in nature), and asked to suggest remedies (where applicable).
- Moderator probing: moderators encouraged participants to expand on their thoughts as they worked through the platform and posed clarifying questions. This probing provided a more comprehensive and in-depth perspective of participants’ experiences using the ASA24.
- Audio and screen recording: participants’ screens were recorded while they completed the survey instruments. Their verbalizations (think-aloud comments and responses to probing questions) were audio recorded using the Adobe Connect Meeting platform or audio recording over the telephone.
- Survey data: a subset of the information entered into the online survey instruments (as described in Section 2.4) was relevant to the usability test, including participant socio-demographic information and administrative and food intake data from the ASA24 to measure task completion and food item count.

#### 2.5.4. Participant Tasks 

**Figure 1:**
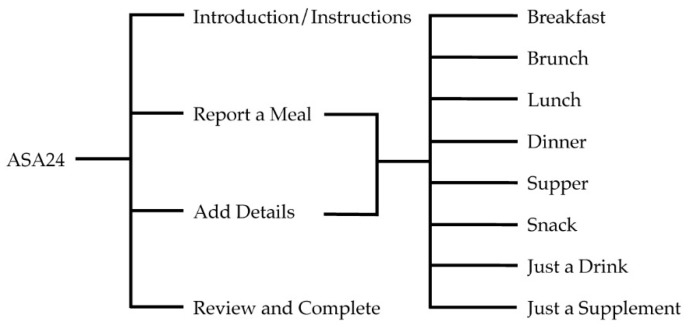
Participant tasks and sub-tasks while completing a dietary recall with the Automated Self-Administered Dietary Assessment Tool (ASA24).

Usability testing requires participants to complete pre-determined and standardized “tasks” that are representative of how they would typically interact with a system. Within the context of this study, the participant’s objective was to complete a dietary recall using the ASA24. The research team defined four tasks and eight subtasks to meet this objective (see Figure 1). All participants had to complete the tasks of reading the introduction (which includes the ASA24 User Orientation), reporting meals (including snacks and drinks), adding details to those meals, and reviewing and completing their entries. The subtasks varied depending on whether or not the individual had consumed the meal in question (i.e., breakfast, brunch, lunch, dinner, supper, snack, just a drink, just a supplement).

#### 2.5.5. Measuring Usability

Measuring usability requires specifying relevant usability metrics. The research team identified four quantitative metrics relevant to the performance of participants using the ASA24. Task success was measured for all three moderation groups, while task time, food item count, and usability issue count could only be quantified for the moderated and semi-moderated groups.

1. Task success, a nominal variable, was defined in three categories: failure to complete the task, completion of the task with moderator assistance, and completion of the task without moderator assistance. The task in this case was completing a dietary recall using the ASA24.
2. Task time, a continuous variable, was defined as the time between a clearly defined start and stop point for each task.
  - Introduction/Instructions: start time when webpage has loaded, stop time when participant clicks “report a meal” button
  - Report a meal: start time when participant has selected a meal from dropdown menu, stop time when participant clicks “finish with this meal” button
  - Add details: start time when participant clicks “add details” button, stop time when ASA24 system loads “review and complete” section
  - Review and complete: start time when webpage has loaded, stop time when ASA24 system dialogue box appears indicating participant has finished their dietary recall
3. Food item count, a nominal variable, was included to account for variability in participants’ dietary intake, which directly affects task performance (i.e., participants who had eaten more food items had more data to enter). This measure was defined as the number of ASA24 output line items in the analytic file for “Items” per meal. For example, if the separate line items for a lunch meal were “fruit, Not Specified (NS) as to type” and “water, municipal” then that meal was assigned a food item count of 2. If there were three separate line items for a meal such as “fruit, NS as to type”, “water, municipal”, and “cheese, cheddar”, then that meal was assigned a Food Item Count of 3.
4. Usability issue count, a nominal variable, was defined as the total number of individual usability issues observed per task. Usability issue identification is defined in detail Section 2.6.2. Generally usability issues are observable events or actions associated with difficulty completing a task.

### 2.6. Data Analysis

Data were collected from the online survey described in Section 2.4, as well as audio and screen recordings from participants in the moderated and semi-moderated groups. The focus of the quantitative and qualitative analysis presented here is only on the data directly relevant to the usability test of the ASA24 (i.e., audio/screen recordings, participant characteristics as reported in the socio-demographic questionnaire, and user data from the ASA24). This section reports the procedures that were used to transform, aggregate, and analyze these data. Data were originally stratified by age (i.e., seniors, non-seniors) and session type (i.e., moderated, semi-moderated, and unmoderated because procedures differed by group). However, performance did not differ by age and, therefore, data are stratified by session type only.

Two members of the research team worked together to first analyze the audio and screen recordings to extract relevant qualitative (i.e., usability issues) and quantitative (i.e., usability metrics) data for subsequent analysis. This audiovisual analysis process is described in Section 2.6.1 and produced a time-stamped record of events and actions for each participant, which was the foundation for subsequent analyses. Qualitative data (e.g., a participant verbalizing that they were unable to find a food item they were searching for) were aggregated and analyzed using thematic analysis, described in Section 2.6.2. Quantitative data (i.e., participant task times, task success, food item count, and usability issue count) were analyzed using descriptive and correlational statistics, described in Section 2.6.3. 

#### 2.6.1. Audiovisual Analysis

Audio and screen recordings from moderated and semi-moderated sessions were analyzed using a software package designed for the behavioural analysis of observational data (Noldus Observer XT (v.14, Noldus Information Technologies, Wageningen, Netherlands). The methodological analysis of audiovisual recordings of users interacting with a “health system” to derive quantitative and qualitative data is described in detail by Mackenzie and Xiao [27]. Central to the process are clear and consistent operational definitions, which analysts assign to events and actions of interest. An operational definition is the formalization of an observable phenomenon so that a researcher can consistently and independently detect it. In this case, the phenomena of interest were usability issues and usability metrics. The two analysts systematically reviewed the audio/screen recordings to identify usability issues (described in Section 2.6.2.) and calculate task times and usability issue counts (described in Section 2.6.3). The two research analysts worked through this process collaboratively and relied on clear and consistent operational definitions to ensure accuracy in the analysis process.

#### 2.6.2. Qualitative Analyses to Identify and Categorize Usability Issues

Usability issues are observable participant behaviours reflecting inefficiency, ineffectiveness, dissatisfaction, or confusion during the use of a system, and an interpretation of the cause of those behaviours relative to the participant’s attempt to complete a task [14]. The research team first identified individual usability issues specific to each participant through audiovisual analysis. These individual usability issues were then subject to thematic analysis, which involved identifying, analyzing, and reporting patterns (themes) within the data [28] to aggregate individual issues into general usability issues. For example, two users may have separately expressed confusion on how to complete an aspect of a task, so these two individual issues would be categorized as the general issue “How to Complete Task Unclear.” To aid in the interpretation of the significance and impact of these general issues, they were then thematically organized based on their relationship to the usability constructs of efficiency, effectiveness, satisfaction, and comprehension.

The two research analysts met regularly to review the data and ensure consistency in interpretations of usability issues. The analysts determined that a point of thematic saturation was reached during the analysis as few to no new usability issues were being observed in the data [29]. In addition, the frequency distribution of usability issues (presented in Section 3.5.) reveals that usability issues in the tails of the distribution were being identified. 

#### 2.6.3. Quantitative Analyses of Usability Metrics

Descriptive statistics were calculated for task time, task success, usability issue count, and food item count. Task success was calculated for all three moderation groups and nominally measured as either failure to complete the ASA24, completion of the ASA24 with assistance, or completion of the ASA24 without assistance. Average task time and individual usability issue count were calculated for the moderated and semi-moderated conditions. The proportional frequency of general usability issues (%) was calculated by dividing the number individual issues counted within a general usability issue category, divided by the total individual usability issue count. For example, 33 out of the 286 individual usability issues were classified as “Question Not Understood”, the proportional frequency of the general usability issue “Question Not Understood” was, therefore, 11.5%. The proportion (%) of participants affected by a general usability issue was calculated by dividing the number of participants that encountered each general usability issue by the total number of participants. Correlations between task time, individual usability issue count, and food item count (from the add details task) were calculated for the report a meal and/or add details tasks using Pearson’s product-moment correlation, with *p* < 0.05 indicating statistically significant correlations. These quantitative analyses were conducted in Microsoft Excel, (2013, Microsoft Corporation, Redmond Washington, WA, USA). 

## 3. Results

### 3.1. Participant Characteristics

Across all moderation groups, participants were mostly female (85.3%) and non-seniors (55.9%). The majority of participants had either only a high school diploma or below (41.1%), or a certificate or diploma below a Bachelor’s degree (including trade certificates and diplomas) (53.7%). While 73.5% of participants were severely or moderately food insecure, the majority still self-reported being in good health or better (79.4%). All participants were from low-income households, which is a criterion for participation in the FMNCP. All participants in the moderated and semi-moderated groups completed the survey using a laptop or desktop computer. It is not known how participants in the unmoderated group accessed the ASA24. Further details can be found in Table 2.

### 3.2. Successful Completion of Dietary Recall Using the Automated Self-Administered Dietary Assessment Tool (ASA24)

In the unmoderated group 94.1% of participants (*n* = 16) failed to fully complete the ASA24. These 16 failures can be characterized as follows:

- Three participants did not initiate either the socio-demographic/health-related survey or the ASA24.
- Two participants initiated the socio-demographic/health-related survey after the 36-h time window had passed, but did not fully complete it or initiate the ASA24.
- Eight participants completed the socio-demographic/health related survey within the 36-h time window, but failed to initiate the ASA24.
- Three participants completed the socio-demographic/health-related survey and initiated the ASA24 within the 36-h time window, but did not fully complete the ASA24 (two of the three participants quit before reporting a meal, and the third participant quit during the add details section).

**Figure 2:**
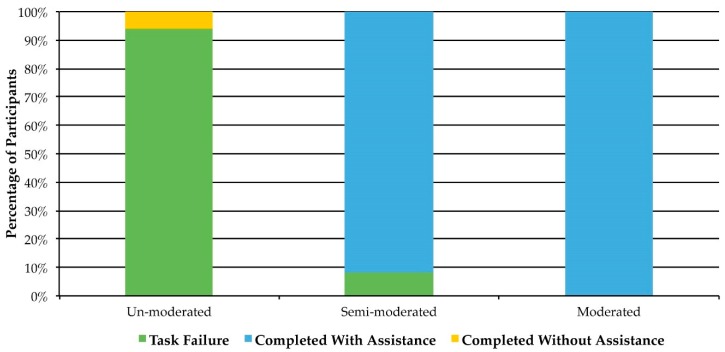
Task success rates for the three groups: unmoderated (*n* = 17), semi-moderated (*n* = 12), and moderated (*n* = 10).

Participants in the unmoderated condition were not observed and there are insufficient data to determine the specific causes of these failures. In the semi-moderated group, 91.7% (*n* = 11) of participants completed the ASA24 with assistance from the moderator (one failed to complete the ASA24), while 100% of the participants (*n* = 10) in the moderated group completed the ASA24 with the assistance of a moderator. In total, only one participant was able to complete the ASA24 without the assistance of a moderator (Figure 2). Because one participant in the semi-moderated group failed to complete the ASA24, quantitative and qualitative data (task completion times, food item count, and usability issues) from this participant were not available for analysis. Additionally, one of the 22 participants in the moderated and semi-moderated group accessed the ASA24 help feature. 

### 3.3. Time to Complete ASA24 Tasks

**Figure 3:**
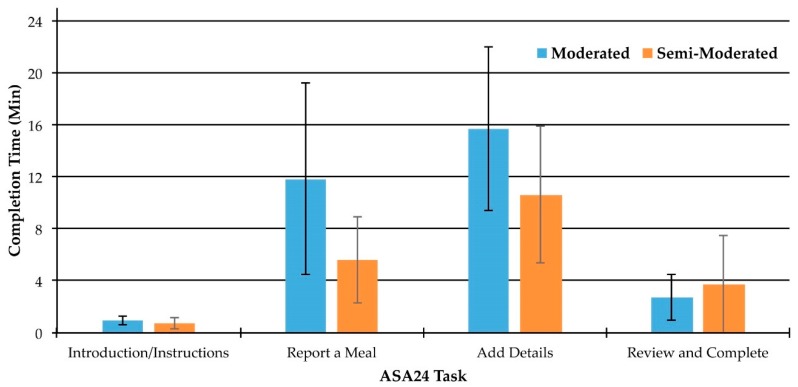
Average task completion times for each task in the moderated (*n* = 10) and semi-moderated (*n* = 11) groups. Error bars represent one standard deviation above and below the mean.

Across both the moderated and semi-moderated groups, the average time it took participants to complete the entire ASA24 (i.e., all four tasks) was 27.4 min (standard deviation (SD) = 12.9). For the moderated group, the average time to complete all tasks was 33.4 min (SD = 14.1). Average task completion time for all four tasks in the semi-moderated group was 21.9 min (SD = 8.7). The report a meal and add details tasks took the longest to complete (Figure 3).

### 3.4. Food Item Count

Food item count provides an indication of differences in the number of steps a participant had to go through to complete the add details task. The average number of food items reported in the add details section in the moderated and semi-moderated groups was 11.5 items (SD = 5.7). The average number of items reported by participants in the moderated group was 12.1 items (SD = 5.6). The average number of items reported by participants in the semi-moderated group was 10.9 items (SD = 5.6).

### 3.5. Usability Issue Frequency

**Figure 4:**
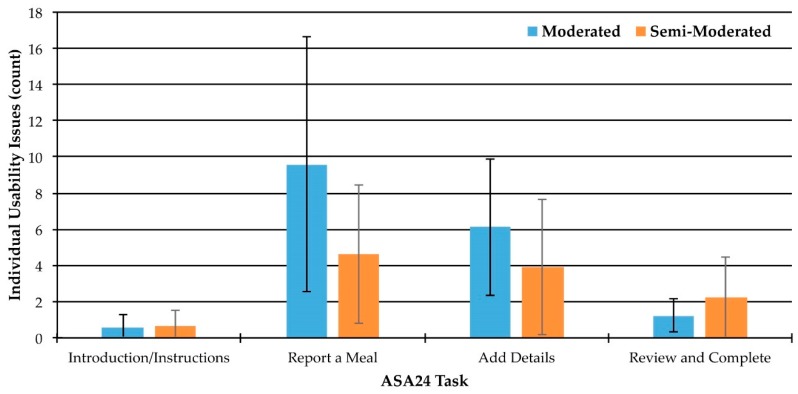
Average number of individual usability issues encountered by participants in the moderated (*n* = 10) and semi-moderated (*n* = 11) groups across all four tasks in the ASA24. Error bars represent one standard deviation above and below the mean.

There were a total of 286 individual usability issues observed in the moderated and semi-moderated groups across all four tasks. There were an average of 13.7 individual usability issues (SD = 8.0) identified per participant. The average number of individual usability issues that participants in the moderated group encountered was 16.6 (SD = 8.7), while those in the semi-moderated group encountered an average of 11.1 (SD = 6.2) usability issues. The majority of individual usability issues were encountered during the report a meal and add details tasks (Figure 4).

**Figure 5:**
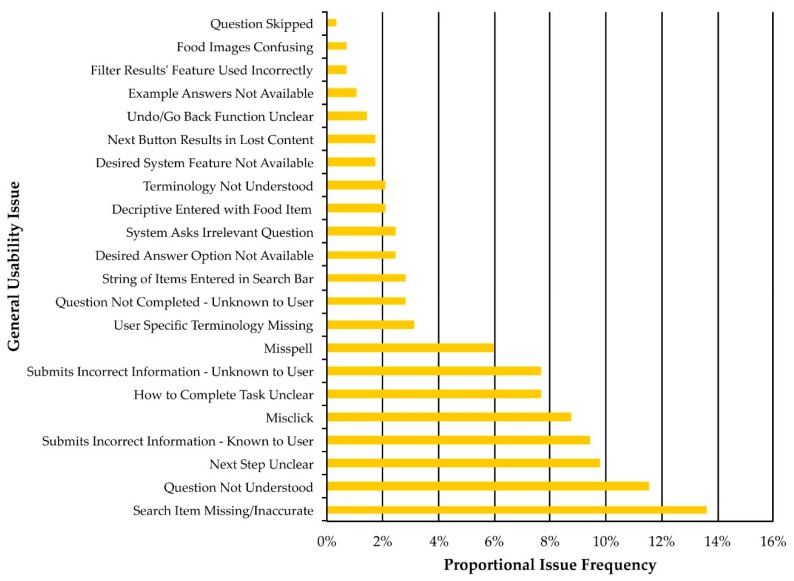
Proportional frequency (%) of each general usability issue across all ASA24 tasks for participants in the moderated (*n* = 10) and semi-moderated (*n* = 11) groups.

The 286 individual usability issues identified were classified within 22 general usability issue categories (see Section 3.7 for definitions and examples). For example, 33 individual issues were identified in which participants did not understand a question asked in the ASA24, all of which corresponded with the general usability issue category of “Question Not Understood”. The proportional frequency of these general usability issues is presented in Figure 5. This analysis revealed that the five most frequent general usability issues were:

1. Search Item Missing/Inaccurate (13.6%)
2. Question Not Understood (11.5%)
3. Next Step Unclear (9.9%)
4. Submits Incorrect Information—Known to User (9.4%)
5. Misclick (8.7%)

**Figure 6:**
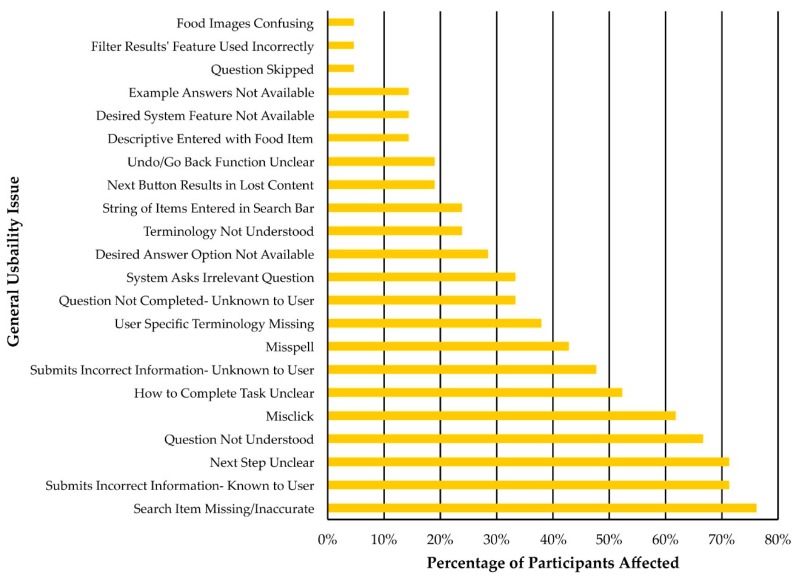
Percentage (%) of participants in the semi-moderated (*n* = 11) and moderated (*n* = 10) groups that were observed being affected by each general usability issue at least once.

The frequency with which participants in the semi-moderated and moderated groups encountered each of the 22 general usability issues is presented in Figure 6. These data indicate the prevalence of each general usability issue within the sample of participants (e.g., out of 21 participants, 14 (66.7%) did not understand an ASA24 question at least once). The five most common general usability issues across all participants were:

1. Search Item Missing/Inaccurate (76.2% of participants, *n* = 16)
2. Submits Incorrect Information—Known to User (71.4% of participants, *n* = 15)
3. Next Step Unclear (71.4% of participants, *n* = 15)
4. Question Not Understood (66.7% of participants, *n* = 14)
5. Misclick (61.9% of participants, *n* = 13)

### 3.6. Correlated Measures

A strong and statistically significant positive correlation was observed between task time and individual usability issue count for the report a meal task, *r* (91) = 0.81, *p* < 0.01. A moderate and statistically significant positive correlation was observed between task time and individual usability issue count for the add details task, *r* (89) = 0.54, *p* < 0.01. A moderate and statistically significant positive correlation was observed between task time and food item count for the add details task, *r* (89) = 0.53, *p* < 0.01. Finally, a moderate and statistically significant positive correlation was observed between food item count and individual usability issue count for the add details task, *r* (89) = 0.45, *p* < 0.01.

### 3.7. Usability Issue Definitions and Examples

The 22 general usability issues and their definitions have been thematically organized based on their relationship to four typical components of usability: Effectiveness, Efficiency, Satisfaction, and Comprehension. 

#### 3.7.1. Usability Issues Related to Effectiveness

Effectiveness refers to a user’s ability to perform a task [30]. Usability issue-related impediments to successful completion of the ASA24 are presented in Table 3. A total of 99 out of the 286 (34.6%) individual usability issues identified relate to the Effectiveness of the ASA24.

#### 3.7.2. Usability Issues Related to Efficiency

Efficiency refers to the effort required by a user to complete a task [30]. In this study, efficiency related to all of the factors that influenced a participant’s ability to complete tasks in a timely and logical way. As opposed to Effectiveness, which refers to whether the user can accomplish a task, Efficiency relates to the effort required to do so. Table 4 presents definitions and examples of usability issues that relate to the ASA24’s Efficiency. A total of 131 out of the 286 (45.8%) individual usability issues related to Efficiency. Table 5 lists food items users were unable to find using the ASA24 search function. Although missing food items reduced the speed of completion, users were still able to complete the task, albeit inefficiently and with inaccurate data.

#### 3.7.3. Usability Issues Related to Satisfaction

Satisfaction refers to a user’s subjective impression of how well a system meets their personal expectations, and can include the desires of the individual with respect to how they would like to use or interact with a system [30]. Table 6 presents a description of the general usability issues that related to user Satisfaction in using the ASA24. A total of 12 out of the 286 (4.2%) individual usability issues related to Satisfaction.

#### 3.7.4. Usability Issues Related to Comprehension

Comprehension refers to whether a user can understand the intended meaning of, and draw accurate conclusions from, the information presented [31]. Comprehension is relevant to text as well as images and diagrams [32]. Usability issues related to a participant’s difficulty or inability to understand questions throughout the ASA24 are presented in Table 7. A total of 44 out of the 286 (15.4%) individual usability issues related to user Comprehension.

## 4. Discussion

This study presents the results of a structured usability test of the ASA24 within a group of low-income, non-University and food insecure adults (85% female, average age 48.2 years) living in British Columbia, Canada. Detailed quantitative data describing how effectively (i.e., task success) and efficiently (i.e., completion time and usability issue count) participants were able to use the ASA24 were collected in real-time and analyzed. In addition, qualitative data were collected and analyzed to explain why individuals performed the way they did. This approach represents a considerable advance over previous studies that evaluated the usability of the ASA24 by relying on users to identify and report retrospectively problematic issues themselves. When considering why researchers, developers, and users of the ASA24 should be concerned about usability, it is helpful to consider the relationship between usability, utility, and usefulness [12]. In the context of the ASA24, utility is the system’s technical capability to enable participants to independently search for, and accurately enter, dietary intake data in a manner that provides the information that researchers require. Usability refers to quantitative and qualitative aspects reflecting how individuals feel and behave while interacting with the ASA24 to access that utility. Usefulness can be considered from two perspectives: (1) how useful is the ASA24 to users who need to enter dietary intake data and (2) how useful is the ASA24 for nutrition researchers who rely on the data it captures. A system with “high usability” makes it easy for users to access its utility and will, therefore, provide data that are more useful for researchers. The purpose of performing a usability test of the ASA24 was to quantify user performance such that usability issues could be identified, quantified and qualitatively described in order to identify opportunities to improve the tool. 

### 4.1. Findings and Recommendations to Enhance Effectiveness of the ASA24

Only one of 17 participants in the unmoderated group successfully completed a dietary recall using the ASA24. Thirteen of the 17 failed to initiate the ASA24 altogether while the other three began the ASA24 but did not complete it. This task failure rate is concerning considering that the unmoderated group was the most representative of how individuals use the ASA24 outside the context of a usability test (i.e. without the support of a moderator). Time pressures may have prevented some participants from initiating the survey within the 36-h time window provided. Others completed the initial socio-demographic/health-related survey, but failed to initiate the ASA24, suggesting that problems logging into the ASA24 and/or participant fatigue led to task failure. Because these participants were not recorded or observed by the moderator it is not possible to identify specific factors that contributed to task failure. Notably, 58% of unmoderated participants were seniors, whereas less than half of those in the other groups were seniors. Among those who initiated, but did not fully complete the ASA24, general usability issues described in Section 3.7.1 can provide a sense of why they may have struggled successfully to complete the ASA24 independently. Of particular note was the common issue of participants being uncertain of the sequence of steps needed to complete a task (Next Step Unclear = 71.4% of participants (15 of 21), How to Complete Task Unclear = 52.4% of participants (11 of 21)). When participants were unclear on how to proceed with a task, it is likely that without the support of a moderator they may have exited the system or potentially entered data incorrectly. 

Further study is needed to understand task failure in individuals using the ASA24 without the assistance of a moderator. Website analytics can provide a useful means of determining problematic sections of the ASA24. For example, a website’s Exit Rate is the percentage of individuals who leave a website from an individual page [14]. The pages of the ASA24 with the highest Exit Rates will likely be associated with task failures. These data could be analyzed by ASA24 developers. 

In the moderated and semi-moderated groups, 21 of 22 participants completed the dietary recall process successfully; however, all of these participants relied on assistance from a moderator at some point to assist them in navigating through usability issues. Whether or not this assistance was necessary for participants to complete a dietary recall is unclear; however, it is clear that the majority of participants benefitted from the support of a moderator. These quantitative data indicate that although the self-administered nature of the ASA24 facilitates data collection, it may ultimately result in low participation rates for groups who encounter frequent usability issues. As the complexity of interaction with a software system increases, it is reasonable to expect that a degree of technical support might be required to support individuals in using that system. None of the participants indicated that they had used the ASA24 before and, therefore, they can be considered novices, demonstrating performance typical of a population of untrained users. Based on these findings, researchers relying on the ASA24 to assess dietary intake in similar populations might expect novice users to require technical support to effectively use the tool for the first time. Thus, availability of on-demand technical assistance may be important to maximize the quantity and quality of data that are collected via the ASA24 and support participant retention.

### 4.2. Findings and Recommendations to Enhance Efficiency of Use of the ASA24

Participants in the present study completed the ASA24 more quickly (~27 min) than has been reported in other studies (~35 min [9,10]) despite the think-aloud procedure and interaction with a moderator. However, these times are comparable to the range reported on the ASA24 website (17–34 min). A strong correlation was observed between task time and food item count in the moderated and semi-moderated groups, with participants consuming an average of 11.5 items daily. Although other studies have not reported item count, an average of 11.5 items daily appears low. Given that the majority of participants had experienced moderate or severe food insecurity during the previous month, this low item count may be related to diminished food access and dietary diversity in our sample, potentially leading to lower ASA24 completion times relative to previous studies. Other characteristics, such as living alone or aging, may also be implicated due to reduced interest in, or capacity to cook. Individuals who are less inclined to cook may consume relatively simple meals with few ingredients. In addition to participant characteristics, the context of completion may also have been influential. Moderators assisted all participants in the moderated and semi-moderated groups at least once. Therefore, it is possible that the task completion times we observed are simply what can be expected when participants have support to use the tool. 

Task time itself is a particularly meaningful usability metric when evaluating repetitive tasks [14]. If task time can be reduced for tasks that most participants must perform repeatedly, gains in efficiency will be achieved. The add details task was the most lengthy to complete for both the moderated and semi-moderated groups. Therefore, supporting users during the add details task will have the greatest impact with respect to enhancing efficiency of use of the ASA24.

Another aspect of how efficiently the ASA24 can be used relates to the overall number and types of usability issues that participants encountered. Participants encountered an average of 13.7 individual usability issues per session. This is concerning considering that usability issues can diminish data quality and present opportunities for task failure or study dropout. The most common general usability issue, experienced by 76.2% of participants (16 of 21), was the inability to find a specific food item. Considering that one of the primary functions of the ASA24 is to enable users to record food intake independently, the prevalence of this usability issue is noteworthy. The inability to locate items was a key source of dietary measurement error. For instance, in the moderated and semi-moderated groups, 71.4% of participants (15 of 21) indicated that they knowingly entered incorrect information at least once, primarily because they were unable to find a specific food item using the search function. Table 5 demonstrates that when this occurred users often selected other items (e.g., substituting steak for a chipotle steak sandwich), used the “I can’t find what I’m looking for” function (the impact of which was having to answer supplemental questions, some of which were irrelevant), or omitted the item entirely. The quantitative impact of this error is unclear, as the current study was not intended to quantify measurement error, but rather to examine its source in order to understand how to mitigate it. However, others have shown that energy intake in adults aged 50–74 years is underestimated by 15–17% on the ASA24 compared to recovery biomarkers, with no difference in mean protein and sodium densities [2]. The current findings can provide complementary data to understand factors that contribute to misreporting of energy intake using the ASA24. 

Additional search-related usability issues concern findings that participants often entered a string of items (e.g., “eggs and toast and water and coffee”) or entered additional descriptive information (e.g., “cold cereal”) into the search bar. Users will increasingly expect any web-based platform they interact with to provide them with “Google-Like” performance, likely with little appreciation for the investment that providing this functionality requires of the developer. Therefore, in addition to adding new food items to the ASA24 database, the intelligence of the ASA24 search algorithm could be improved to recognize plural forms of food items (e.g., the system returned a result for “taco” but not “tacos”) and to suggest potential matches when multiple words or descriptors are entered into the search bar. 

Usability issues related to misclicking and misspelling were also prevalent. This could be a reflection of users not knowing what to do in the system, contributing to data errors or task failures (e.g., exiting the system). Additionally, entering an incorrectly spelled food item into the system can lead the user through additional irrelevant questions. This contributes to increased task times, and potentially decreased user satisfaction. This inefficiency could be quantified in subsequent usability tests using the metric of “lostness” [33] by comparing the number of steps an individual performs to the minimum number of steps possible. This calculation would enable the impact of misclicks to be more thoroughly understood from an efficiency perspective. Some degree of user error (whether typos or accidental clicks) is outside the control of designers or researchers, however others can be addressed through relatively simple design changes (e.g., the visual differentiation or clarification of a button or text field’s function). 

### 4.3. Findings and Recommendations to Enhance Satisfaction with the ASA24

Satisfaction in use is perhaps one of the most easily conceptualized aspects of usability; presumably, if someone is happy using a system then it is likely usable. Previous studies have asked participants to self-report their satisfaction with the ASA24, finding that the majority of participants had a favourable view of the system [8,34,35,36]. The current study examined specific system features that contributed to user satisfaction or dissatisfaction. Given these different outcomes, ability to compare our findings with those of others was limited. However, similar themes emerged in our study compared to others’, including frustrations about the time involved in completing a recall and how to proceed to the next step, not understanding how to use the search function, and not being able to find food items [10]. Users who feel that a system is not designed to allow them to use it intuitively are unlikely to want to continue to use that system. One design approach to address satisfaction is to provide users with shortcuts that allow them to duplicate repetitive actions [37]. Future usability tests could compare how easy individuals expect a task to be before attempting it (expectation score) to how easy they found it after completing it (experience score). When users expect a task to be easy to complete but then find it difficult (i.e., expectation score is much higher than the experience score), it is very likely to lead to dissatisfaction [38]. This approach would help prioritize potential design changes specifically to improve satisfaction. 

### 4.4. Findings and Recommendations to Enhance Comprehension of the ASA24

The second most frequent general usability issue identified was Question Not Understood, which accounted for 11.5% of all issues and was experienced by 66.7% of participants (14 of 21). Often, when participants were confused, it was observed that they had only partially processed the elements of the question being asked (e.g., mistaking which particular food item they were being asked about) or that they had not understood the specific words or phrasing of the question itself (e.g., general comments such as “I don’t know what this means.”). An individual who does not understand what the ASA24 is asking them to do, or who becomes confused by terminology or imagery, is likely to have difficulty using the tool to provide accurate information. A general approach to enhancing comprehension is to match the user’s mental model (e.g., ensuring language doesn’t exceed the user’s reading level, presenting visuals in a way that match the way the user consumes the food, requesting measurements of quantity that align with the user’s method of measurement, etc.). This can be challenging given the wide variability in individuals’ mental models, particularly for a tool such as the ASA24 that is intended to be used broadly across multiple populations. Two alternate design strategies that might be considered would be to: (1) allow users to customize the ASA24 to match their mental model (e.g., switching units from metric to imperial, alternative visual depictions of foods, customizable reading levels for text presentation), or (2) standardize the tool but ensure that it has been optimized to meet the needs of the majority of users through comparative comprehension testing. 

### 4.5. User Characteristics and Usability Testing

Participants were recruited from a population participating in a nutrition coupon program and as such were primarily low-income, low- to mid-educated, and food insecure adults, many of whom were seniors. Older adults and those of a lower socioeconomic status may have lower computer literacy compared to the general population [39] and it is, therefore, possible that some of the usability issues identified here may be particular to this sample. As we did not assess computer literacy, it is unclear whether this was an issue in our sample. Darajeh and Singh [40] have summarized design recommendations to enhance usability for those with lower computer literacy, including creating simple layouts with limited clutter, providing user guides, reducing the use of complex terminology, creating simple navigation paths, using similar functions for different tasks, and including descriptive text for tool use. Dietary inequities and strategies to support optimal dietary patterns among older adults are significant concerns worldwide, and thus our findings can inform nutrition studies among these priority populations. 

### 4.6. Strengths and Limitations

One of the strengths of the mixed methods approach was the ability to both quantify and qualitatively describe usability issues, providing a much more comprehensive and in-depth perspective of the usability of the ASA24. Moreover, the think-aloud procedures and inductive nature of the analyses enlarged the scope of investigation beyond researchers’ pre-determined questions and response options to uncover novel usability issues. The qualitative analysis had a sufficient sample size to reach a point of thematic saturation and we are, therefore, confident that the analysis uncovered the most salient usability issues in this particular sample. 

The validity of a usability test is partially dependent on creating test conditions that reflect the actual conditions under which a user interacts with a system. The presence of a moderator may create a Hawthorne or Observer Effect in which participant behavior changes due to being observed, participants are overly reliant on assistance from a moderator to complete a task, or experience heightened sensitivity to usability issues. Having participants think-aloud may also create additional cognitive demand and thereby alter task performance. These factors could all contribute to a test scenario in which reported usability issues and performance metrics are not perfect representations of those that would have been encountered during actual use.

In addition, the definition of task failure as used in this study is specific to our methodological design. Participants were not given multiple attempts to complete the ASA24; if they failed to complete it upon their first attempt this was recorded as a task failure. However, the tool does allow participants to complete a dietary recall in multiple attempts. This study also looked at the ASA24 when used in combination with a socio-demographic/health-related survey. This survey was administered before the ASA24, which may have influenced participant behavior. For example, completing surveys prior to the ASA24 may create additional fatigue, affecting motivation to complete the ASA24. Finally, participants in the unmoderated group may have failed to complete the ASA24 for reasons unrelated to its usability (e.g., interruptions, variability in motivation). Researchers interested in using the ASA24 in a similar population should be aware of these potentially high participant drop-out rates. 

### 4.7. Help Documentation and Training in Relation to Usability.

The ASA24 does provide a help guide for users and Best Practices information for researchers, in addition to the help feature embedded in the ASA24 [41]. However, just one of the 22 participants observed in the moderated and semi-moderated groups accessed the ASA24’s help feature. Help functions do not, however, improve usability because they place the onus of efficient and effective system use on the user, increasing their workload rather than making system changes to enhance usability. Moreover, users often do not read support materials [42]. One of the main benefits of conducting usability testing, or designing usable systems, is that the process will reduce costs associated with training and customer support [43]. 

## 5. Conclusions

This study demonstrates how the usability of the ASA24 affects its usefulness for a particular group of users entering dietary intake data as well as for researchers studying that information. One of the primary benefits of the self-administered nature of the ASA24 is the relative ease (for researchers) with which dietary intake data can be collected. However, the results of this study highlight important limitations of this self-administered approach. Task success data reveal that the vast majority of individuals in our sample had difficulty independently using the ASA24 without the support of a moderator. Moreover, the frequency and nature of usability issues identified suggest that information was often entered inaccurately, as 71.4% of participants knowingly misentered dietary information at least once. Other key usability issues encountered were related to difficulties using the search function, not understanding certain questions, uncertainty regarding how to proceed to the next step, and misclicks. It is not clear to what extent our findings are specific to our sample of primarily non-university educated adult females (average age 48.2 years), with a low household income. We expect that other groups may encounter similar challenges, albeit perhaps at a lower frequency. Our findings can help to understand how the ASA24 can be improved to make it more intuitive and simple for individuals from a wider variety of populations to use, thereby enhancing the accuracy of dietary intake reporting. The following recommendations are intended to address key usability issues users encountered.

### 5.1. Key Recommendations for Designers of the ASA24

1. Improve the intelligence of the ASA24 search algorithm such that it is responsive to user search behaviors including pluralization, synonyms, multi-item searches and misspelling.
2. Remove irrelevant questions (e.g., asking the user where they obtained the ingredients for their tap water).
3. Use intuitive language and terminology (e.g., instead of a button that says “add details”, change so it reads “next”).
4. Create a demonstration video that shows participants how to use the ASA24 and that addresses key usability issues they may encounter (i.e., those that developers cannot remedy through design changes).

### 5.2. Key Recommendations for Researchers Using the ASA24

1. Technical assistance should be available on demand to assist participants who are using the ASA24, particularly for first time users.
2. Strategies for effective use of the ASA24 should be explained in advance.
3. Request and allow participants to report retrospectively that they entered information that was not accurate (e.g., if they could not find an exact match for a particular food) and allow them to report what the actual food was.

## Figures and Tables

**Table 1:** Moderator-participant interactions and data sources in the three session types.

	Moderator Probing	Participant Think-Aloud	Audio and Screen Recording	Survey Completion Data
Unmoderated	×	×	×	✓
Semi-Moderated	×	×	✓	✓
Moderated	✓	✓	✓	✓

**Table 2:** Participant characteristics according to moderation group.

	Moderated (*n* = 10)	Semi-Moderated (*n* = 12)	Unmoderated ^1^ (*n* = 12)	Total (*n* = 34)
**Age (years), mean ± standard deviation (SD)**	43.9 ± 20.3	43.5± 17.7	57.2 ± 18.4	48.2 ± 19.0
**Seniors, *n* (%)**	4 (40.0)	4 (33.3)	7 (58.3)	15 (44.1)
**Non-seniors, *n* (%)**	6 (60.0)	8 (66.7)	5 (41.7)	19 (55.9)
**Gender, *n* (%)**				
Women	7 (70.0)	10 (83.3)	12 (100.0)	29 (85.3)
Men	3 (30.0)	2 (16.7)	0 (0.0)	5 (14.7)
**Self-reported health, *n* (%)**				
Excellent	0 (0.0)	1 (8.3)	0 (0.0)	1 (2.9)
Very Good	0 (0.0)	1 (8.3)	3 (25.0)	4 (11.8)
Good	8 (80.0)	7 (58.3)	7 (58.3)	22 (64.7)
Fair	1 (10.0)	3 (25.0)	2 (16.7)	6 (17.7)
Poor	1 (10.0)	0 (0.0)	0 (0.0)	1 (2.9)
**Education level** **, *n* (%)**				
Less than high school	1 (10.0)	3 (25.0)	4 (33.3)	8 (23.5)
High school	5 (50.0)	1 (8.3)	0 (0.0)	6 (17.6)
Trade certificate/diploma	1 (10.0)	3 (25.0)	0 (0.0)	4 (11.8)
Certificate or diploma below Bachelor’s	3 (30.0)	4 (33.3)	7 (58.3)	14 (41.9)
Bachelor’s degree	0 (0.0)	0 (0.0)	1 (8.3)	1 (2.9)
Above Bachelor’s degree	0 (0.0)	1 (8.3)	0 (0.0)	1 (2.9)
**Employment Status, *n* (%)**				
Full-time employment	0 (0.0)	2 (16.7)	2 (16.7)	4 (11.8)
Part-time employment	1 (10.0)	1 (8.3)	1 (8.3)	3 (8.8)
Unemployed: Looking for work	1 (10.0)	0 (0.0)	1 (8.3)	2 (5.9)
Unemployed: Not looking for work	0 (0.0)	4 (33.3)	2 (16.7)	6 (17.7)
Student	1 (10.0)	0 (0.0)	0 (0.0)	1 (2.9)
Retired	3 (30.0)	1 (8.3)	5 (41.7)	9 (26.5)
Other	4 (40.0)	4 (33.3)	1 (8.3)	9 (26.5)
**Receiving Social Assistance or Welfare, *n* (%)**	5 (50.0)	7 (58.3)	5 (41.7)	17 (50.0)
**Immigration Status, *n* (%)**				
Native Canadian				
Immigrant: English-speaking country	9 (90.0)	12 (100)	10 (83.3)	31 (91.2)
Immigrant: Non-English-	1 (10.0)	0 (0.0)	2 (16.7)	3 (8.8)
speaking country	0 (0.0)	0 (0.0)	0 (0.0)	0 (0.0)
**Marital Status, *n* (%)**				
Single, never married	1 (10.0)	4 (33.3)	4 (3.3)	9 (26.5)
Living common-law	3 (30.0)	1 (8.3)	0 (0.0)	4 (11.8)
Married	1 (10.0)	2 (16.7)	1 (8.3)	4 (11.8)
Separated	2 (20.0)	2 (16.7)	0 (0.0)	4 (11.8)
Divorced	3 (30.0)	1 (8.3)	6 (50.0)	10 (29.4)
Widowed	0 (0.0)	1 (8.3)	1 (8.3)	2 (5.9)
Did not answer	0 (0.0)	1 (8.3)	0 (0.0)	1 (2.9)
**Food Insecurity Status, *n* (%)**				
Food secure	1 (10.0)	1 (8.3)	3 (25.0)	5 (14.7)
Marginally food insecure	2 (20.0)	2 (16.7)	0 (0.0)	4 (11.8)
Moderately food insecure	3 (30.0)	3 (25.0)	7 (58.3)	13 (38.2)
Severely food insecure	4 (40.0)	6 (50.0)	2 (16.7)	12 (35.3)
**Access to Internet at Home, *n* (%)**				
Yes	8 (80.0)	11 (91.7)	12 (100.0)	31 (91.2)
No	2 (20.0)	0 (0.0)	0 (0.0)	2 (5.9)
Did not answer	0 (0.0)	1 (8.3)	0 (0.0)	1 (2.9)
**Preferred Internet Access Method ^2^, *n* (%)**				
Computer		7 (58.3)		7 (58.3)
Smartphone		3 (25.0)		3 (25.0)
No Preference		2 (16.7)		2 (16.7)
Did not answer		1 (8.3)		1 (8.3)

^1^ Not all participants in the unmoderated group completed the sociodemographic questionnaire; ^2^ Some participants chose both computer and smartphone as preferred methods to access the internet.

**Table 3:** Descriptions and examples of usability issues related to Effectiveness.

Usability Issue	Definition	Example/Application
Question not completed—unknown to user	User does not answer questions because they do not realize they are mandatory.	Occurred most often in the “frequently forgotten foods” section in which some participants did not realize they had to respond to each question and not just the questions they found applicable (although the system later forced them to provide an answer).
Undo/go back function unclear	It is unclear to user how to undo an action or revert back to a previous screen.	Occurred when participants wanted to edit a meal that had already been submitted as well as general confusion whilst navigating through the tool.
Next step unclear	User is confused about how to proceed, particularly when transitioning from one task to the next.	The language on some buttons did not match the participant’s expectations (e.g., the button to begin adding details read “add details” but participants expected to click a button labeled “next”). Buttons were also often located “below the fold” meaning that participants had to scroll down to see them.
How to complete task unclear	User knows what goal they want to accomplish but are unsure how to do so.	Many participants were unsure how to begin entering their meals or had other questions about how to do so. For example, “I made pizza last night so do they want me to put in pizza dough, sauce, cheese?”
Submits incorrect information—unknown to user	User misinterprets task and enters incorrect information but do not realize that they have made an error.	Occurred most often when participants were entering food items. Most of these issues were clear to the moderators. However, when moderators suspected that participants had entered something incorrectly without realizing it, they asked the participant to clarify what they had intended to do. For example, entering “coconut” instead of “coconut sugar”, but believing they had entered coconut sugar.
Question skipped	User chooses not to answer a question.	Occurred when participants decided not to answer a question that was asked (although the system later forced them to provide an answer).
Descriptive entered with food item	User enters either the size, amount, number of food items, or other adjectives in the search bar along with the item itself.	Occurred when entering foods in the search bar (the ASA24 does not recognize adjectives). For example, searching for “cold cereal” instead of “cereal”.
String of items entered in search bar	User enters several food items into the search bar, not realizing that items must be entered individually.	Occurred when entering foods in the search bar. For example, entering “eggs and toast and water and coffee” into the search bar.

**Table 4:** Descriptions and examples of usability issues related to Efficiency.

Usability Issue	Definition	Example/Application
User-specific terminology missing	Users search for food items in terms familiar to them that the system did not recognize.	Occurred when entering foods in the search bar. For example, searching for “Palm Bay” instead of the system recognized “vodka cooler”.
System asks irrelevant question	User is asked questions that are not relevant based on answers they have previously given.	Occurred most frequently during “add details” tasks. For example, asking participants where they obtained the ingredients for their tap water.
Search item missing/inaccurate	Users search for food items in common language but the system returns either no results or inaccurate results.	Occurred when entering foods in the search bar. See Table 5 for a list of items not found in the system and items participants substituted when this occurred.
Next button results in lost content	Users proceed to the next task without correctly submitting the previous task. After realizing the error and reverting back, content previously entered has been lost.	Occurred most often when participants selected “finish with this meal” (similar to the function of a “next” button) before correctly adding all food items to the meal.
Submits incorrect information—known to user	Users deliberately enter incorrect information as determined via the participant’s verbalization that they were doing so.	Occurred in situations where the participant felt it was too much work to enter the information accurately, because they weren’t sure what the correct information was, or because they wanted to enter the information accurately but did not know how to do so. For example, “This is assuming I only had one burger so I am just going to say 3 patties because realistically I had 3 burgers.”
Misclick	Users click in a location that is different from where they need to click to accomplish a task.	Occurred often when the participants clicked “next”, which took them to the “add details” task when they had not finished reporting all of their meals.
Misspell	User makes a spelling mistake when searching for or entering a food item.	Occurred when entering foods in the search bar or via free text.
Filter results feature used incorrectly	User misinterprets the list of food items returned from search and/or uses it incorrectly.	Occurred when participants reviewed the list of food items returned from search. For example, the ASA24 offers functionality to filter the search results. One participant interpreted those filter options as ingredients for the food items they were entering, which resulted in confusion.

**Table 5:** Food items searched by the user that did not return the desired result, and the resulting food item selected by the user.

Food Item Searched by User	Food Item(s) Selected by User
Apricots	Could not/did not enter item
Greek yogurt	Fruit-flavoured yogurt
Coconut cookie	Sugar cookie
English muffin	Multigrain bread
Cherries	Fruit salad
Coconut sugar	Coconut
Green smoothie	Fruit smoothie
Spring water	Bottled water
Gluten free bread	Whole wheat bread
Garlic powder	Could not/did not enter item
French vanilla cream	Half and half cream
Rice bowl	Could not/did not enter item
Chocolate covered almonds	Candy
Salt	Could not/did not enter item
Pepper	Could not/did not enter item
Chipotle steak sandwich	Steak
Tacos	Taco
Onion (as addition to hamburger)	Could not/did not enter item
Tomato (as addition to hamburger)	Could not/did not enter item
Dill pickle (as addition to hamburger)	Could not/did not enter item

**Table 6:** Descriptions and examples of usability issues related to Satisfaction.

Usability Issue	Definition	Example/Application
Desired answer option not available	Response options did not reflect the desired response options of the user	Occurred most frequently when answering whether the amount of food eaten was “usual”, “much more than usual”, or “much less than usual”. Many participants felt that “more than usual” or “less than usual” would have provided a more accurate picture of the quantity eaten.
Desired system feature not available	System features do not allow users to report intake accurately in a convenient way	Many participants wanted an easier way to accurately represent their diet than what was offered by the system. For example, many wanted to report water consumed intermittently throughout the day as opposed to entering each instance of water consumption individually.

**Table 7:** Descriptions and examples of usability issues related to Comprehension.

Usability Issue	Definition	Example/Application
Question not understood	Users do not understand the meaning of a question	Participants often misunderstood questions. This occurred most frequently during the “add details” task, when the system switched from requesting details about one item, to requesting details about another, but participants did not notice that they were now being asked to add details of a new food item.
Terminology not understood	Users do not understand the terminology contained within a question	Participants often did not understand specific words used in certain questions. For example, when users were asked to report meals, both “dinner” and “supper” were response options which led to user confusion.
Example answers not available	Users are not presented with the same answer options as presented in the question	There was a mismatch between the way some questions were phrased and the available response options. For example, one participant was asked “String cheese: was it regular, reduced fat, low fat, non-fat, or something else?” and the answer options in the dropdown menu were “part skim”, “other”, and “don’t know”.
Food images confusing	Food images are not representative of items actually consumed	Occurred when food images did not resemble the items that participants had eaten. For example, one participant who had sliced their zucchini lengthwise became confused when the system showed a zucchini that was sliced width-wise.
